# Fiber-Optic Liquid Level Sensing by Temperature Profiling with an FBG Array

**DOI:** 10.3390/s18082422

**Published:** 2018-07-25

**Authors:** Francesco Barone, Alessandro Signorini, Laurent Ntibarikure, Tiziano Fiore, Fabrizio Di Pasquale, Claudio J. Oton

**Affiliations:** 1 Scuola Superiore Sant’Anna, TeCIP Institute; Via Giuseppe Moruzzi 1, 56127 Pisa, Italy; fabrizio.dipasquale@santannapisa.it (F.D.P.); c.oton@santannapisa.it (C.J.O.); 2 Infibra Technologies; Via Scornigiana 42, 56121 Ospedaletto, Italy; a.signorini@infibratechnologies.com; 3 Baker Hughes, a GE Company, Nuovo Pignone Tecnologie; Via Felice Matteucci 2, 50127 Florence, Italy; Laurent.Ntibarikure@bhge.com (L.N.); fiorti@hotmail.it (T.F.)

**Keywords:** FBG, optical fiber sensor, liquid level sensor, temperature, distributed temperature sensors

## Abstract

We describe a fiber-optic system to measure the liquid level inside a container. The technique is based on the extraction of the temperature profile of the fiber by using a fiber Bragg grating (FBG) array. When the temperatures of the liquid and the gas are different, the liquid level can be estimated. We present a physical model of the system and the experimental results and we compare different algorithms to extract the liquid level from the temperature profile. We also show how air convection influences the temperature profile and the level of estimation accuracy. We finally show dynamic response measurements which are used to obtain the response time of the sensor. Turbomachinery monitoring is proposed as one possible application of the device.

## 1. Introduction

Liquid level sensing is an ubiquitous and important measurement in many industries (chemical, oil and gas, automotive, food). The request for automated systems and very accurate and reliable process control increases the need for advanced level measurement systems [1]. Different technologies used for liquid level sensing include flotation, hydrostatic pressure, electrical impedance, ultrasound reflection, radio and light reflection [2,3].

Some applications may require flexible sensors which can be adapted to different shapes of container which can be irregular. Multiple measurements along a container wall can also monitor the tilt angle.

In previous years, the use of optical fiber sensors has spread in the sensor market. Their reliability has increased and their use is becoming common in many industries, especially for temperature and strain measure. In particular, Fiber Bragg Gratings (FBG) have become common due to their capability of sensing both strain and/or temperature at many points along a fiber [4,5]. These sensors are immune to electromagnetic interference. Tthey are chemically inert, robust to harsh environments, suitable for explosive atmosphere and intrinsically passive. For these reasons, turbomachinery monitoring is one specific application which could greatly benefit from fiber-optic sensing technology.

FBGs are typically unsensitive to the presence of a surrounding liquid, because the light is usually confined to the core of the fiber. However, fibers can be made sensitive to the presence of a liquid, for example, by using long-period gratings (LPGs) [6,7], laterally-etched FBGs [8], tilted FBGs [9], coreless fibers [10,11], etc. On the other hand, the presence of a liquid can be indirectly estimated by measuring a different magnitude, for example, Diaz et al. [12] used an FBG on a diaphragm to measure the hydrostatic pressure and calculate the liquid level. In reference [13], hydrostatic thrust was exploited to design a cantilever with an embedded FBG strain sensor to compute the liquid level. Bending loss or inter-fiber coupling can also be sensitive to the presence of a liquid, making a liquid sensor exploit these effects [14,15,16]. Chen and coworkers used an optical power source to actively heat the fiber, measure the thermal response of the fiber using an FBG array and to detect the level [17]. This technique does not require a temperature difference between the liquid and the gas, but it has a limited spatial range due to the fact that the heating takes place at a single point. Other fiber optical sensor solutions to liquid level measure can be found in reference [18].

In this paper, we estimate the liquid level from the temperature profile of an FBG array. If there is a temperature difference between the liquid and the gas above, one can estimate the level from the position of temperature change. Conditions of a liquid permanently at a different temperature to the surrounding environment are frequent in many industrial applications, for example, cooling liquid sumps. Furthermore, as the fiber only measures temperature, it can be protected from the environment inside a metal tube, making the sensor suitable for harsh environments.

The paper is divided as follows: first, the mathematical model of the sensor system is described in detail in Section 2.1, highlighting crucial parameters that influence the measure. Three kinds of algorithms for level estimation are proposed in Section 2.2: the first and the second ones are empirical, and the third is based on model fitting. Then, the experimental setup is described in Section 2.3. The experiments are described in Section 2.4, and their results are shown in Section 3, where we show different temperature profiles under different conditions of liquid level and air speed. The average measurement error is within ±2 mm, lower than half of the FBG spacing. We also show dynamic measurements which are used to estimate the response time of the system. Finally, the results are discussed in Section 4.

## 2. Materials and Methods

### 2.1. Sensor System Mathematical Model

In order to understand and fit the results, a mathematical model of the system was developed. The model allows the calculation of the expected temperature profile which will facilitate the extraction of the liquid level from the experimental data.

First it was assumed that on the horizontal plane, XY, the temperature inside the fiber and the metallic protection is constant. Furthermore, the heat loss coefficient of the tube with the liquid was assumed to be infinitely high, so the temperature of the immersed FBG can be approximated with the liquid temperature.

The heat distribution variation in the volume of the tube is expressed by the heat equation:

(1) $$\rho c_{p}\frac{\partial u(x,y,z,t)}{\partial t}-\nabla \cdot \left(k\nabla u\left(x,y,z,t\right)\right)={\dot{q}}_{v},$$

where ρ is the mass density; $c_{p}$ is the specific heat capacity; *k* is the thermal conductivity of the metal; ${\dot{q}}_{v}$ represents the volumetric heat sources or losses; and u(x,y,z,t)=T(x,y,z,t)+c is the temperature distribution function inside the considered volume with an arbitrary additive constant, *c*, which is set to $-T_{air}$ to make calculations simpler. $T_{air}$ is the temperature of the air which is assumed to be constant.

**Figure 1:**
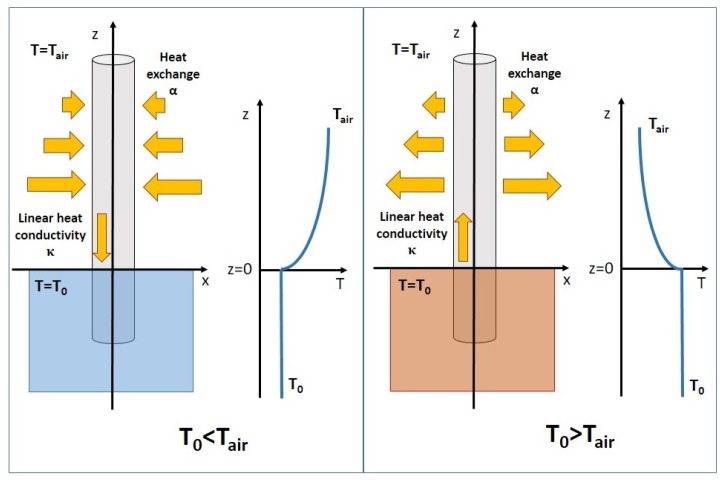
System mathematical model: the sensor temperature is assumed to be constant below the water level and tends to the air temperature following an exponential curve with a decay rate of $\sqrt{\frac{\alpha }{\kappa }}$.

The temperature profile along the fiber is due to two main phenomena [19] (see Figure 1):

- the thermal conductivity along the metallic protection of the fiber, expressed by −∇·(k∇u(x,y,z,t)) in Equation (1);
- the thermal loss of the metallic protection with the outer air, included in the term ${\dot{q}}_{v}$ of the equation.

At steady state, the time derivative has to be equal to zero. Assuming the temperature is constant on the horizontal plane, XY, the partial derivative $\frac{\partial }{\partial x}$ and $\frac{\partial }{\partial y}$ must be zero. Thus, the one-dimensional heat equation becomes

(2) $$\kappa \frac{d^{2}u\left(z\right)}{dz^{2}}+{\dot{q}}_{v}\left(z\right)=0,$$

where κ is the linear thermal conductivity which, for a tube, is equal to

(3) $$\kappa =kA=k\pi (R_{ext}^{2}-R_{int}^{2}),$$

where *A* is the area of the metallic section of the tube; $R_{ext}$ is the external radius; $R_{int}$ is the internal radius; and *k* is the thermal conductivity of the metal.

The model considers that the thermal loss from the tube to the air takes place through convection exchange, where ${\dot{q}}_{v}\left(z\right)$ is assumed to be proportional to the temperature difference:

(4) $${\dot{q}}_{v}\left(z\right)=-\alpha u\left(z\right),$$

where α is the heat convective dissipation coefficient of the tube with the air, and *u* is the temperature difference between the tube (T(z)) and the air:

(5) $$u\left(z\right)=T\left(z\right)-T_{air}.$$

By replacing (4) and (5) in Equation (2), we obtain the following second-order differential equation:

(6) $$\kappa \frac{d^{2}u\left(z\right)}{dz^{2}}-\alpha u\left(z\right)=0.$$

Assuming the *z*-axis is aligned to the axis of the vertical tube with the positive direction upwards and the level of the water at *z* = 0, we impose the boundary conditions $u\left(0\right)=T_{0}-T_{air}$ and u(∞)=0; thus, the analytical solution for z≥0 is:

(7) $$T\left(z\right)=\left(T_{0}-T_{air}\right)e^{-\sqrt{\frac{\alpha }{\kappa }}z}+T_{air}.$$

Figure 1 shows the expected temperature profile. The decay rate of the exponential temperature profile along the axes of the sensor depends on thermal linear conductivity of the fiber shield, κ, and the heat exchanges with the air, α. The level of detection from the temperature profile is easier when the decay rate is higher, thus with an higher α and a lower κ.

The thermal conductivity along the tube is known as it only depends on the tube’s geometry and the thermal conductivity of the material. Instead, α is more unpredictable, as it depends on environmental conditions of the air—the more the air is exchanged, the higher the number becomes. The influence of α on the liquid level estimation measure was measured during the tests, as described in Section 2.4.

### 2.2. Level Detection Algorithms

An automatic measurement requires a robust algorithm. In this work, three detection algorithms were compared. The first two are empirical methods (MaxDer and ThCross), while the third one exploits the physical model (ModFit).

#### 2.2.1. Maximum Derivative Algorithm (MaxDer)

The expected temperature profile (see Section 2.1) has the maximum derivative corresponding to the liquid level. This suggests the first simple algorithm:

1. Measure THE temperature for each FBG sensor
2. Compute the spatial derivative
3. Find where the derivative is maximum

**Figure 2:**
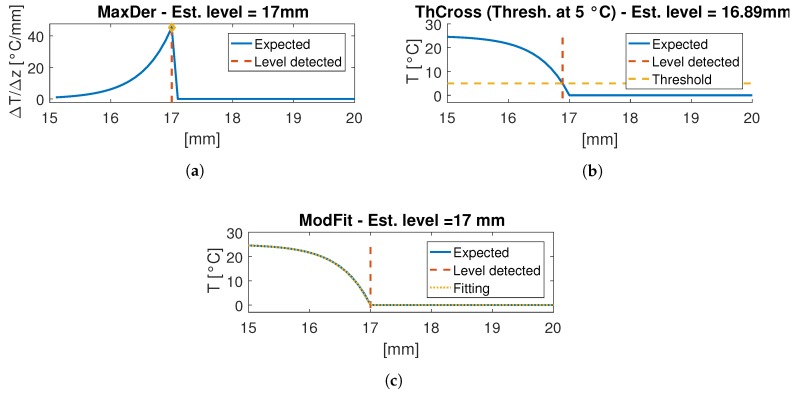
Algorithms tested on simulated data. The simulated level is at 17 mm. (**a**) MaxDer; (**b**) ThCross. An offset has to be added to reach the correct level. (**c**) ModFit.

This kind of algorithm has a resolution that is equal to the distance between the center of one FBG to the next one if the array is mounted vertically, is not curved and FBGs are equally spaced. Such a resolution can be sufficient for an array with an high sensor density, but it does not exploit the information of each sensor. Moreover the derivative is generally an algorithm that is sensitive to noise in measures; therefore, it could require an average filter to obtain a smooth measurement. Furthermore, it is not robust to unexpected phenomena, because it could modify the local temperature profile and detect a completely wrong level. Figure 2a shows how the MaxDer algorithm estimates the liquid level from the spatial derivative. The main advantage of this algorithm is the implementation simplicity.

#### 2.2.2. Threshold Crossing-Based Algorithm (ThCross)

The second algorithm relies on the choice of a threshold and it consists of the following steps:

1. Measure the temperature for each FBG sensor
2. Estimate the temperatures of the liquid, ${\tilde{T}}_{0}$, and the air, ${\tilde{T}}_{air}$
3. Interpolate the measured temperature profile with a spline
4. Choose a temperature threshold between ${\tilde{T}}_{0}$ and ${\tilde{T}}_{air}$
5. Find where the threshold crosses the temperature profile

Figure 2b shows the expected temperature profile crossing a threshold at 5 °C: the level can be approximated with the abscissa of the crossing point (dashed red line). The estimation of ${\tilde{T}}_{0}$ and ${\tilde{T}}_{air}$ may be the measures of fixed sensors (one inside the water and one outside) or they can be kept constant. A small accuracy error is accepted and it is lower as much as the threshold is near ${\tilde{T}}_{0}$ rather than ${\tilde{T}}_{air}$, but it is more robust to temperature deviations from the profile expected from the model. This accuracy error can be compensated by fixing an offset value and adding that to the estimated level.

#### 2.2.3. Parametric Model Fitting (ModFit)

The third algorithm exploits all information about the temperatures and the model. It is outlined by the following steps:

1. Measure the temperature for each FBG sensor
2. Fit the temperature profile with the following parametric function (extracted from the expected temperature profile in Equation (7))
  (8) $$u\left(z\right)=\left\{\begin{array}{cc} ae^{-b(z-l)}+c & \mathrm{if}\;z>l\\ c & \mathrm{if}\;z\leq l \end{array}\right.,$$
  where *a*, *b c* and *l* are the parameters.
3. Compute the physical values from parameters:
  (a) $T_{0}=c$;
  (b) $T_{air}=a+c$;
  (c) $\alpha =\kappa b^{2}$ where κ is the linear thermal conductivity which is known for stainless steel;
  (d) The estimated level is the parameter *l* of the fitted function.

This is the algorithm that fits the temperature profile to the model presented in the previous section. Figure 2c shows an example of liquid level estimation which calculates the level exactly by definition. This algorithm is expected to be more reliable compared to the other algorithms, because it is the result of a fit to a physical model of the system.

### 2.3. Sensor System Description

The experimental tests were performed in order to validate the proposed measurement of the liquid level, where the gas above it has a different temperature. The measurement consists of several simultaneous temperature measurements through an optical fiber with an array of FBGs inscribed on it. Knowing the temperature profile along the fiber and its location inside the container is sufficient to estimate the level of the liquid in thermal equilibrium.

To protect the fiber from the harsh environment and from the manipulation during deployment, the FBG array was inserted into a stainless steel tube (thermal conductivity of 20 W/mK).

**Figure 3:**
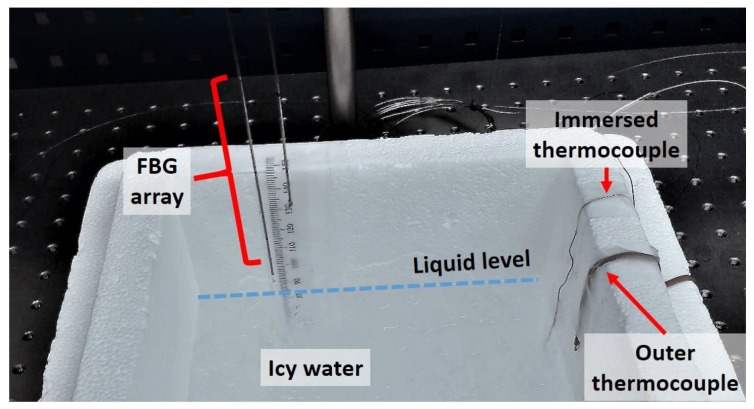
System setup: a polystyrene container was filled with icy water, and an FBG array was moved vertically in order to simulate the level change. Two thermocouples measured the water and air temperatures.

The tube has to be thermally conductive to allow temperature to propagate radially to the fiber. Nevertheless, a conductive material will also conduct temperature axially along the fiber; thus, the temperature profile along the fiber will be smoother, and the level will become less detectable. If there is a big enough temperature difference between the liquid and the gas, the level detection can be estimated. In our setup (see Figure 3) the shield was a tube with an external radius of 0.4 mm and an internal radius 0.2 mm.

An array of 10 FBG was used; each FBG was 3 mm long and the center-to-center spacing was 5 mm. The total length of the array was 48 mm. The last FBG was 10 mm from the fiber tip. The FBG reflection wavelengths at 0 °C were uniformly distributed from 1533 nm to 1560 nm (wavelength spacing was 3 nm). Each FBG was individually calibrated; the average wavelength–temperature relationship was equal to 10.5 pm/K, and the standard deviation among gratings was 0.27 pm/K. Along the sensing length, the fiber had no coating; thus, we can discard any coating-related hysteresis effect in its thermal response.

The metallic shield used was semi-rigid, keeping the optical fiber straight. The shielded array was partially immersed in icy water inside a container of a thermal insulator material (polystyrene), in order to keep the water temperature as constant as possible. The array was set vertically. In order to simulate level changes, the FBG array was moved up and down, instead of adding or removing water. The FBG array was monitored with an FBG interrogator (Hyperion si255 of Micron Optics in Atlanta, USA), set to a 1 kHz sampling frequency with 10 averages, leading to an acquisition rate of 100 Hz. Two thermocouples measured the water temperature at the bottom of the container and the air temperature at 20 mm above the water level to verify if there were any environmental changes.

### 2.4. Experiments

The sensor system (sensors and algorithms) was tested to evaluate its performance. The acquired temperatures were also used to validate the mathematical model and quantify the influence of α on the measure. In order to generate a fixed temperature of 0 °C in the water, a mixture of 50% water and 50% ice was used.

The first experiment was a calibration and its main aim was to measure the system’s accuracy. The sensorized tube with the FBGs array inside was mounted on a support whose height was manually set. The sensor temperatures were acquired throughout the experiment, visualized on a chart and stored on a PC.

The first position was at the maximum height. The second position was set with the tip of the tube touching the water level and other height regulations followed until the 10 FBGs were below the water level. Between one regulation and the next one, the temperature steady state was checked on a real-time chart. During regulation, the support heights were manually measured with a tape measure and recorded in order to calculate each FBG distance from the water level. FBG temperatures were acquired at seven height steps. Each height step was about 5 mm.

In the second and third experiments, new datasets were acquired in a forced convection regime using a compressed air gun. Pressure values were fixed at 0.25 and 0.5 bars, respectively. Air speeds were measured with a vane anemometer (Urceri Mini Thermo-Anemometer), in each case, obtaining wind speeds of $s_{1}=4.8$ and $s_{2}=7.1$ m/s respectively. For both wind speeds, air speed temperatures were acquired at four different height steps.

The last tests were done to test the dynamic response of the system. In these tests, we measured the response to a step-like full immersion and a full extraction. The displacement of the array took less than 0.5 s in each case. These tests were done with an air speed of $s_{0}=0$ m/s.

Data was post-processed using Matlab and Curve Fitting Toolbox. The fitting algorithm used was the Least Absolute Residual method. The threshold, $T_{th}$, for the ThCross method was set at $T_{th}=T_{min}+0.30\times \left(T_{max}-T_{min}\right)$. Then, the measurement was corrected, adding an offset to make the mean error equal to zero. The offset depends on α, so three different offsets were used for each air speed.

## 3. Results

**Figure 4:**
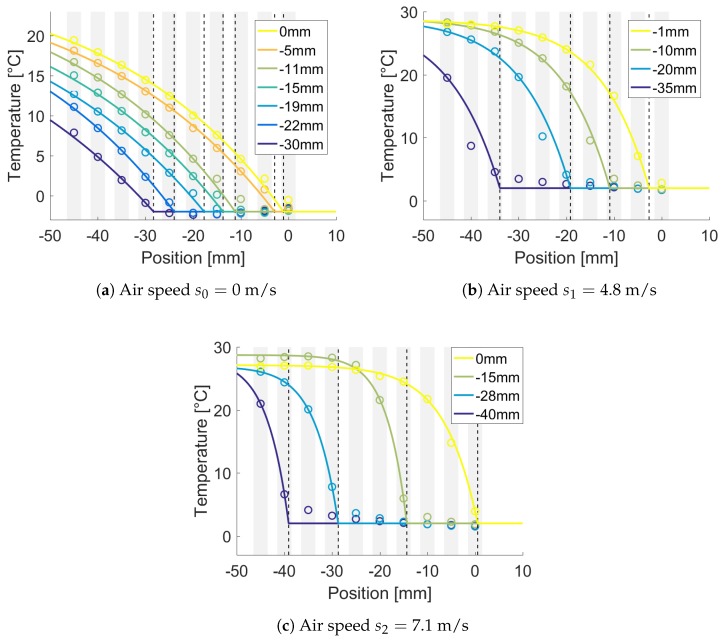
Model validation by comparison with measured data. The plots show the measured data (circles) and the fitted model for each level. The dotted vertical lines show the levels estimated by the ModFit algorithm. The gray vertical stripes show the Fiber Bragg Gratings (FBG) positions.

The acquired temperatures are shown as circles in Figure 4a with no forced convection. Temperature profiles from the fitted model are also shown. The 10-th FBG was the nearest to the icy water. The same plots were obtained for the two level of air speed, $s_{1}$ and $s_{2}$ (see Figure 4b,c). The mean $R^{2}$ for the different air speeds was higher than 0.95 ($R_{s_{0}}^{2}=0.995$; $R_{s_{1}}^{2}=0.986$; $R_{s_{2}}^{2}=0.995$).

The mean value and standard deviation for the fitted parameters were as follows: 1/b = 39 mm ± 3.3 for $s_{0}$, 1/b = 10.2 mm ± 0.3 for $s_{1}$ and 1/b = 5.2 mm ± 1.2 for $s_{2}$. Thus, the estimated α values were 50 mW/mK ± 0.8, 72.5 mW/mK ± 3.7 and 312 mW/mK ± 112 respectively.

The expected temperature profiles and the estimated levels shown in Figure 4 were obtained by fitting the model as described in Section 2.2.3.

The three algorithms were tested on data acquired during the three measurements.

**Figure 5:**
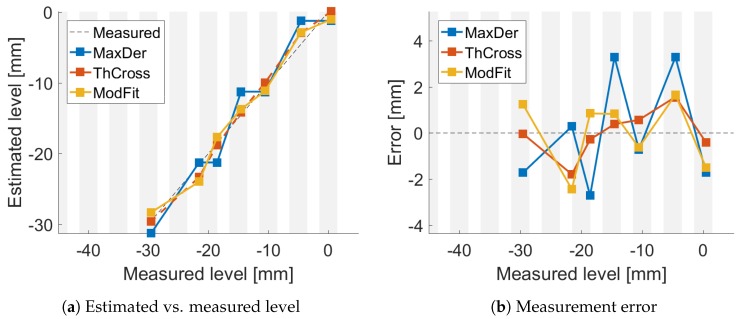
Comparison of algorithms without moving air.

**Figure 6:**
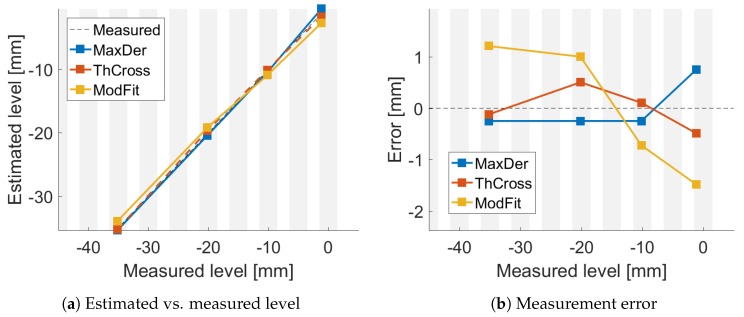
Comparison of algrithms with air speed at $s_{1}$.

**Figure 7:**
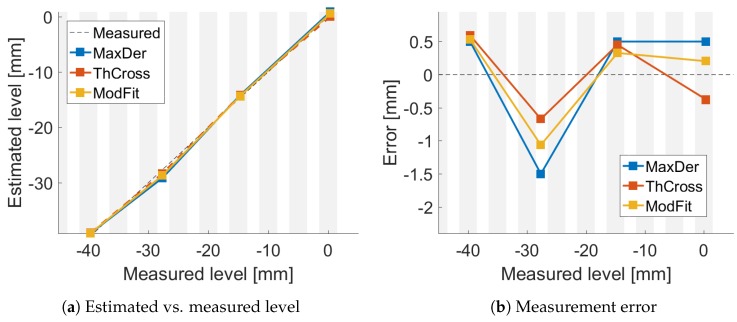
Comparison of algorithms with air speed at $s_{2}$.

The estimated levels were plotted versus the measured ones and are shown in Figure 5a, Figure 6a and Figure 7a. The resulting errors are shown in Figure 5b, Figure 6b and Figure 7b for each method. The gray vertical bars show the FBG positions and width, compared to the measured level. Thus, it is possible to highlight where the FBGs were compared to the liquid level.

**Figure 8:**
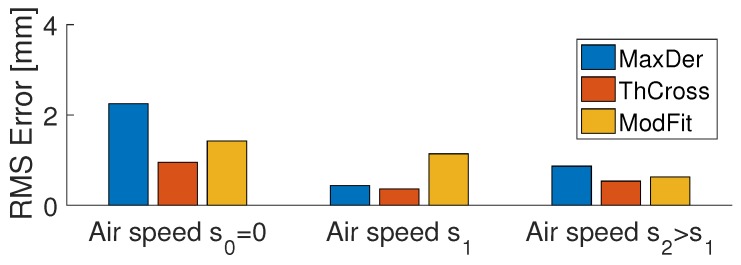
Error comparison: the bars show the average error for each air speed condition and for each estimation algorithm.

The bar chart summarizes the performance of the sensor system with the three different estimation methods (see Figure 8). The bars show the root mean squared errors (RMSE) for different air speeds. We achieved better accuracy than the FBG spacing (5 mm). The average absolute error for any estimation method is lower than half of FBG spacing.

**Figure 9:**
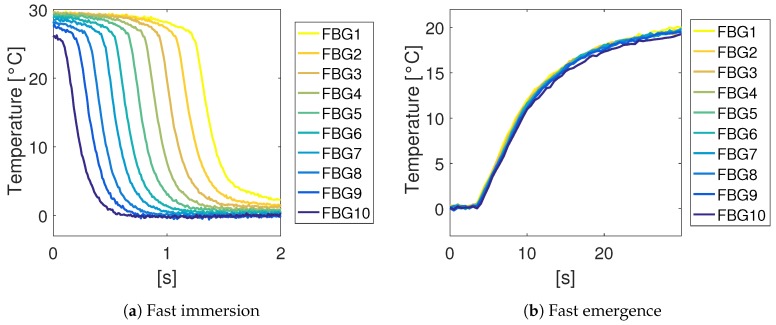
Temperature dynamic response to fast immersion (**a**) and emergence (**b**). The exponential fits gave time constants of $\tau_{I}$ = 0.12 s (immersion) and $\tau_{E}$ = 8 s (emergence).

The results of the dynamic tests are shown in Figure 9. The temperature curves were fitted with an exponential function in order to estimate the dynamic time constant. The time constant during emergence $\tau_{E}$ was about 8 s, while during immersion, $\tau_{I}$ was 0.12 s.

## 4. Discussion

Figure 4a shows good agreement between the data and the temperature mathematical model ($R^{2}\geq 0.95$). Even though the liquid temperature was not homogeneous and drifted during the different experiments, the liquid level was estimated with a reasonable accuracy using the three methods, as shown in Figure 5, Figure 6 and Figure 7.

From the performance bar chart (see Figure 8), we can see that the MaxDer method achieved better results with an increased α (i.e., with forced convection; air speed greater than zero). The ThCross method with offset calibration achieved the best results. A high α (high air speed) increased the performance of ThCross, but this was due to the calibration with a constant α. If α varies with time but the offset is kept constant, an additional error is expected. Finally, the ModFit algorithm performance was almost unaffected by α variations and it kept its RMSE below 2 mm. As the liquid level estimation comes from the result of a fit, instead of comparing the sensitivity or resolution, we can use the obtained accuracy of the measurement to compare it with the resolution reported in other works. For example, Ricchiuti et al. [6] achieved 2 mm of liquid level resolution in a 10 cm range of sensing refractive index change with LPG, while our system had no cross-sensitivity with the refractive index of the liquid.

Regarding the dynamic response measurements, we reported time constants of $\tau_{I}$ = 0.12 s for immersion and $\tau_{E}$ = 8 s for emergence. This difference of almost two orders of magnitude was expected as the dynamics of the immersion were dictated by the thermal exchange between the liquid and the tube which was much faster than the air–tube heat exchange which determines the emergence time constant. This means that when the liquid level rises, the system responds much faster than when the liquid level decreases. In particular, a time response of 8 s could be a limiting parameter when fast level variations are expected. However, in terms of monitoring a slowly-varying liquid level, the device could be useful in many applications. When faster responses are needed, it is expected that the response time could be reduced by different approaches, such as (i) introducing forced air convection; (ii) coating the tube with a product which prevents the formation of droplets on the surface, which were observed in the experiments and probably have a negative influence on the speed of the sensor; or (iii) removing or reducing the thickness of the protection metal tube.

## 5. Conclusions

We have demonstrated the feasibility of liquid level estimation by temperature profile sensing with an FBG array. We proposed a simple physical model of the system, and we found good agreement with the experiments. Three algorithms for level estimation were proposed and tested; all of them reached an RMSE lower than half of the FBG spacing. The proposed technique is applicable when there is a certain temperature difference between the liquid and the gas. This work showed successful results for a temperature difference of 25 °C and an estimated heat exchange coefficient between 50 and 312 mW/mK. Its applicability to smaller temperature differences would depend on the air convection conditions and thus would require specific testing. Finally, we reported a response time of 0.12 s for immersion and 8 s for emergence, the latter limiting its application to situations in which the liquid level varies slowly. With all of these considerations, turbomachinery monitoring is one sector in which this technology can find scenarios of application.
